# Draft Genome Sequences of Eight Canadian Citrobacter braakii and Citrobacter freundii Strains

**DOI:** 10.1128/MRA.00273-19

**Published:** 2019-07-03

**Authors:** Marc-Olivier Duceppe, Beverley Phipps-Todd, Catherine Carrillo, Hongsheng Huang

**Affiliations:** a Canadian Food Inspection Agency, Ottawa Laboratory – Fallowfield, Fallowfield, Ontario, Canada; b Canadian Food Inspection Agency, Ottawa Laboratory – Carling, Carling, Ontario, Canada; University of Maryland School of Medicine

## Abstract

Citrobacter braakii and Citrobacter freundii are Gram-negative opportunistic pathogens associated with many infectious diseases, including septicemia, in humans and animals. Here, we report the draft genome sequences of seven C. braakii strains and one C. freundii strain isolated from Canadian wastewater treatment facilities.

## ANNOUNCEMENT

Citrobacter braakii and Citrobacter freundii, two of the 15 species in the genus Citrobacter, are Gram-negative coliform bacteria in the Enterobacteriaceae family (http://www.bacterio.net/citrobacter.html). They can be found in soil, water, sewage, and the intestinal tracts of animals and humans. They are opportunistic pathogens, causing infections of the urinary, gastrointestinal, and pulmonary systems, as well as blood infections, often nosocomial in nature; they are a major cause of neonatal meningitis (1, 2). Citrobacter freundii generally exhibits high antimicrobial resistance to penicillin and third-generation cephalosporins (3), which harbor a β-lactam ring.

This article reports the draft genome sequences of seven C. braakii strains and one C. freundii strain isolated from pre- or posttreated samples collected at three Canadian wastewater treatment facilities. Strains HH1, HH5, HH6, HH8, HH10, and HH11 were isolated using a Compendium of analytical methods of Health Canada method (MFHPB-19) (4) for the isolation of Escherichia coli. Strains HH7 and HH9 were isolated using a Compendium of analytical methods of Health Canada method (MFLP-75) (5) for the isolation of Salmonella species. All isolates were confirmed to be Citrobacter at the genus level by a combination of the API 20E biochemical test (bioMérieux, Canada, Inc.), Biolog (carbohydrate analysis), and Sherlock (fatty acid analysis) (Midi, Inc., Newark, DE, USA) microbial identification systems (Table 1). The isolates were further identified as *C. braakii* or C. freundii by the API 20E test. Species identifications were confirmed by comparing the draft genome sequences reported here (Table 1) with the reference genome of each species.

**TABLE 1:** Sequencing and assembly metrics for the strains used in this study

Strain	Species	Assembly size (bp)	No. of contigs	*N*_50_ value (bp)	GC content (%)	No. of CDS	Coverage (×)	No. of reads	Assembly accession no.	SRA accession no.
HH1	*C. braakii*	4,824,576	18	803,039	52.1	4,638	99	1,643,354	GCA_002923765	SRR6664068
HH5	*C. braakii*	4,873,670	21	757,685	52.0	4,664	63	673,281	GCA_004331575	SRR8643105
HH6	*C. braakii*	5,128,900	54	254,837	51.9	4,958	91	1,064,342	GCA_004331585	SRR8643104
HH7	*C. braakii*	4,825,329	15	803,041	52.1	4,577	90	848,821	GCA_004331545	SRR8643107
HH8	*C. braakii*	5,401,766	77	235,111	51.9	5,236	71	819,903	GCA_004331535	SRR8643106
HH9	*C. braakii*	5,001,420	26	435,834	52.1	4,811	67	790,355	GCA_004331635	SRR8643109
HH10	*C. braakii*	5,326,488	84	210,999	51.8	5,222	70	852,877	GCA_004331445	SRR8643108
HH11	C. freundii	5,449,209	97	183,552	51.7	5,396	79	1,031,234	GCA_004331565	SRR8643110

Isolates were kept at −80°C in tryptic soy broth with 25% glycerol before sequencing. Genomic DNA was extracted from overnight cultures grown at 37°C on tryptic soy agar using the DNeasy blood and tissue DNA purification kit (catalog number 69504; Qiagen, Inc., Toronto, Ontario, Canada) and quantified using a Qubit 3.0 fluorometer (Life Technologies). Sequencing libraries were constructed using Nextera XT DNA sample preparation kits (Illumina, Inc., San Diego, CA), and the quality of libraries was analyzed using a Bioanalyzer instrument (Agilent Technologies, Santa Clara, CA, USA). Libraries were sequenced with a MiSeq platform (Illumina, Inc.) using 300-bp v3 reagent kits.

Sequencing adapter sequences and low-quality bases were trimmed (BBduk), and overlapping pairs were merged (BBMerge) using the BBTools v37.68 software suite (https://jgi.doe.gov/data-and-tools/bbtools/). The remaining reads were then assembled *de novo* with Unicycler v0.4.4 (6) running SPAdes v3.11.1 (7) and then polished with Pilon v1.22 (8). Other programs used by Unicycler were BLAST v2.7.1+ (9), Bowtie 2 v2.3.4 (10), and SAMtools v1.6 (11). The parameters used for each step of the assembly pipeline are detailed at https://github.com/duceppemo/bacteria_genome_assembly. Gene predictions and annotations were performed using the NCBI Prokaryotic Genome Annotation Pipeline (12). Sequencing and assembly metrics are summarized in Table 1. Antimicrobial resistance genes were detected using ResFinder v3.1.0 (13), with default parameters.

The median total length, protein count (number of coding sequences [CDS]), and GC content of the *C. braakii* genome assemblies in the NCBI are 5.1 Mb, 4,822, and 52.1%, respectively. Similarly, the median values of the seven *C. braakii* strains presented here are 5.1 Mb, 4,672, and 52.0%, respectively. The median total length, protein count (number of CDS), and GC content of the C. freundii genome assemblies on NCBI are 5.3 Mb, 4,991, and 51.7%, respectively, which are similar to those (5.499 Mb, 5,396, and 51.7%, respectively) of the C. freundii strain analyzed in this study (Table 1). All isolates were predicted to be resistant to β-lactam antibiotics.

### Data availability.

This whole-genome shotgun project has been deposited at DDBJ/ENA/GenBank under the accession numbers PQWA00000000, SJSJ00000000, SJSI00000000, SJSH00000000, SJSG00000000, SJSF00000000, SJSE00000000, and SJSD00000000 for strains HH1, HH5, HH6, HH7, HH8, HH9, HH10, and HH11, respectively. The versions described in this paper are versions PQWA01000000, SJSJ01000000, SJSI01000000, SJSH01000000, SJSG01000000, SJSF01000000, SJSE01000000, and SJSD01000000, respectively. The Illumina raw sequencing reads are available in the NCBI Sequence Read Archive through the accession numbers found in Table 1.
